# Spatial Distance Correlates With Genetic Distance in Diffuse Glioma

**DOI:** 10.3389/fonc.2019.00676

**Published:** 2019-07-30

**Authors:** Evan D. H. Gates, Jie Yang, Kazutaka Fukumura, Jonathan S. Lin, Jeffrey S. Weinberg, Sujit S. Prabhu, Lihong Long, David Fuentes, Erik P. Sulman, Jason T. Huse, Dawid Schellingerhout

**Affiliations:** ^1^Department of Imaging Physics, The University of Texas MD Anderson Cancer Center, Houston, TX, United States; ^2^Graduate School of Biomedical Sciences, The University of Texas MD Anderson Cancer Center UTHealth, Houston, TX, United States; ^3^Department of Radiation Oncology, NYU Langone School of Medicine, New York, NY, United States; ^4^Department of Translational Molecular Pathology, The University of Texas MD Anderson Cancer Center, Houston, TX, United States; ^5^Medical Scientist Training Program, Baylor College of Medicine, Houston, TX, United States; ^6^Department of Bioengineering, Rice University, Houston, TX, United States; ^7^Department of Neurosurgery, The University of Texas MD Anderson Cancer Center, Houston, TX, United States; ^8^Department of Radiation Oncology, The University of Texas MD Anderson Cancer Center, Houston, TX, United States; ^9^Department of Pathology, The University of Texas MD Anderson Cancer Center, Houston, TX, United States; ^10^Departments of Neuroradiology and Cancer Systems Imaging, University of Texas MD Anderson Cancer Center, Houston, TX, United States

**Keywords:** glioma, genomics, epigenetics, stereotactic biopsy, medical image analysis, radiomics, imaging genomics, radiologic-pathologic correlation

## Abstract

**Background:** Treatment effectiveness and overall prognosis for glioma patients depend heavily on the genetic and epigenetic factors in each individual tumor. However, intra-tumoral genetic heterogeneity is known to exist and needs to be managed. Currently, evidence for genetic changes varying spatially within the tumor is qualitative, and quantitative data is lacking. We hypothesized that a greater genetic diversity or “genetic distance” would be observed for distinct tumor samples taken with larger physical distances between them.

**Methods:** Stereotactic biopsies were obtained from untreated primary glioma patients as part of a clinical trial between 2011 and 2016, with at least one biopsy pair collected in each case. The physical (Euclidean) distance between biopsy sites was determined using coordinates from imaging studies. The tissue samples underwent whole exome DNA sequencing and epigenetic methylation profiling and genomic distances were defined in three separate ways derived from differences in number of genes, copy number variations (CNV), and methylation profiles.

**Results:** Of the 31 patients recruited to the trial, 23 were included in DNA methylation analysis, for a total of 71 tissue samples (14 female, 9 male patients, age range 21–80). Samples from an 8 patient subset of the 23 evaluated patients were further included in whole exome and copy number variation analysis. Physical and genomic distances were found to be independently and positively correlated for each of the three genomic distance measures. The correlation coefficients were 0.63, 0.65, and 0.35, respectively for (a) gene level mutations, (b) copy number variation, and (c) methylation status. We also derived quantitative linear relationships between physical and genomic distances.

**Conclusion:** Primary brain tumors are genetically heterogeneous, and the physical distance within a given glioma correlates to genomic distance using multiple orthogonal genomic assessments. These data should be helpful in the clinical diagnostic and therapeutic management of glioma, for example by: managing sampling error, and estimating genetic heterogeneity using simple imaging inputs.

## Introduction

Gliomas are thought to be genetically heterogeneous within a single specimen, as manifested by spatial morphological diversity observed on imaging. Genetic analyses are increasingly important in the delineation of glioma subgroups with distinct clinical behavior, as evidenced by the strong influence of genomic classifiers in the WHO 2016 grading system (1). Previous work has shown that biopsies taken from non-representative regions of tumor can produce errors in histopathological grading (2), and while modern image guidance may improve histopathological accuracy (3, 4), there are strong suggestions from the literature that genetic heterogeneity may also be underrepresented by standard surgical sampling (5, 6). Additionally, we know from work in other tumors that genomic signatures can vary depending on regional sampling (7).

If, therefore, molecular heterogeneity varies as a function of location in space, then it is reasonable to *hypothesize* that such variability might correlate with physical (Euclidean) distance between biopsy sites. A formal relationship between Euclidean and molecular distance *per se* has not (to our knowledge) been described for glioma. In this study, we seek to address this *gap in knowledge*, both qualitatively and with a quantitative statistical assessment.

To do so, we obtained multiple sets of stereotactic biopsies in previously untreated glioma patients, carefully noted the physical coordinates of each sample, and calculated the Euclidean distances between each pair of samples within a single tumor. Multidimensional genomic analysis was then performed on each sample, and distinct measures of genomic distance were derived from: (1) mutation number, (2) copy number variation, and (3) the extent of CpG island methylation. We found that in each case, meaningful and positive correlations were present between Euclidean and genetic distance.

## Methods

### Biopsy Collection

Our study retrospectively analyzed glioma tissue samples collected as part of an IRB approved, HIPAA-compliant clinical trial protocol (NCT03458676). All subjects gave written informed consent in accordance with the Declaration of Helsinki. Biopsies were collected from previously untreated adult (>18 years old) patients with primary glioma immediately prior to tumor resection. Each patient underwent pre-surgical MRI within 3 days prior to craniotomy. During surgery, two or more image-guided biopsies were collected from each patient. The biopsy locations were chosen based on one or more findings in pre-operative MRI including contrast enhancement, reduced diffusivity, or increased cerebral blood flow. This approach mimics clinical workflow and targets areas likely to harbor malignant tumor tissue. Samples were collected using either a side-cutting, Nashold-type, image-guided biopsy needle (0.9 mm width and 10 mm side port) or by image-registered surgical biopsy forceps based on surgeon preference and patient anatomy. Samples were collected before tumor resection in order to minimize brain shift and we estimate the variance in the distance measurements based on the recorded image coordinates to be <2 mm. Tissue samples were immediately placed on ice for transport to a pathology lab where the tissue was frozen in OCT until analysis.

### Biopsy Euclidean Distance

At the time of the biopsy collection, we recorded the image coordinates of the instruments using the surgical navigation software. The distance between separate biopsy sites *i* and *j* is calculated by the Euclidean distance of the captured 3D coordinates (*x, y, z*): $d_{ij}=\sqrt{{\left(x_{i}-x_{j}\right)}^{2}+{\left(y_{i}-y_{j}\right)}^{2}+{\left(z_{i}-z_{j}\right)}^{2}}$ (Figure 2). When possible for needle biopsies, the “shallow” and “deep” ends of the cylindrical specimens were divided. These specimens were analyzed separately with a distance of 5 mm assigned to the two parts of the divide sample, based on the needle geometry. The exact geometry is illustrated in Supplementary Figure S2.

### DNA Extraction

Using light microscopy, each sample was microscopically confirmed to be comprised of tumor before DNA extraction. Percent wise quantification was not attempted due to the small amount of tissue in each sample. DNA was extracted from frozen biopsies and matched normal white blood cells (WBCs) using QIAamp DNA Mini Kit (Qiagen), and DNA concentrations were measured with Qubit fluorometer (Thermo Fisher Scientific).

### Whole-Exome Sequencing

Between 200 and 1,000 ng of DNA were used for enrichment of all exonic fragments with SureSelect Human All Exon V6 (Agilent Technologies), followed by massively parallel sequencing on HiSeq4000 platform (Illumina) using 75-bp paired-end option. For the validation of somatic mutations identified by the HiSeq platform, custom PCR primer panels corresponding to the mutations were made with Ion AmpliSeq Designer. The libraries were prepared with Ion AmpliSeq Library Kit Plus (Thermo Fisher Scientific) according to the manufacture's protocol, and then subjected to Ion Proton sequencing (Thermo Fisher Scientific). Where available, we estimated tumor cellularity and ploidy using the whole-exome data and the sequenza (8). This confirmed the tumor content identified microscopically.

### Mutation Count Genetic Distance

With the whole-exome sequencing (WES) fastq files, we used the BWA-MEM (9) software for read mapping. With the bam files, we used MuTect2 (10) software to call genetic variations between tumor samples and blood control samples and used ANNOVAR (11) to annotate the specific mutations (pseudocode provided on Github).^1^ We then filtered the resulting mutations based on the five following criteria: (1) mutations must be located in exonic regions; (2) mutation function must be frameshift deletion, frameshift insertion, non-synonymous SNV, stopgain or stoploss; (3) reference read count, the read having same base call as reference, must be ≥10; (4) alterative read count, the reads detected as mutations, must be ≥8; and (5) the alternative read frequency must be ≥0.1. These filters ensure the mutations are real, not statistical artifacts, and that they likely lead to molecular tumor changes such as reduced expression levels, truncated proteins, or errors in DNA transcription and translation. The mutation count genetic difference between samples from the same tumor in one patient was measured using the Jaccard distance (12).

$$\begin{array}{l} d_{Jaccard}=\frac{\sum {\left(P_{i}-Q_{i}\right)}^{2}}{\sum P_{i}^{2}+\sum Q_{i}^{2}-\sum \left(P_{i}*Q_{i}\right)} \end{array}$$

Where *P*_*i*_ and *Q*_*i*_ are the alternative allele frequency for the *i*th mutation. The mutation count genetic distance between samples is small when the gene mutations are present in both samples and maximum when the sets of mutations are disjoint. Pearson correlation coefficients were calculated between the number of genes/mutation genetic distance and the Euclidean distance for all available biopsy pairs.

### Copy Number Variation Genetic Distance

Copy number variations (CNV) for paired biopsies were obtained using WES data with CNVKit (13), which has high CNV calling accuracy (14) and can infer information in uncovered intron regions. With the segmentation information for each biopsy, we combined all break points available from all biopsies, created a list of CNV events, and assigned the corresponding log2 ratio value to each event and each biopsy. The CNV distance was calculated using the Canberra distance

$$\begin{array}{l} d_{Canberra}=\sum \frac{\left|P_{i}-Q_{i}\right|}{P_{i}+Q_{i}} \end{array}$$

Where *P*_*i*_ and *Q*_*i*_ are the log ratio values of the first and second samples at event *i* (12) between paired biopsies from the same patients. The Canberra distance is effectively the L_1_ distance but scaled at each value by the average signal of the samples. This normalizes the differences so that samples with larger relative absolute difference will be a greater distance apart under this metric. So, CNV distance is a measure of the total amount of DNA variation between samples. Also note that the distance between a sample and itself is zero. Pearson correlation coefficients were calculated between the CNV distance and the Euclidean distance for each biopsy pair.

### Methylation Distance

DNA was subjected to bisulfite conversion with EZ DNA Methylation-Gold Kit (Zymo Research), and analyzed for methylation profiling using Infinium Methylation EPIC Beadchip and iScan (Illumina). We evaluated differences in DNA methylation as a way to quantify the epigenetic distance between samples and investigate correlation with physical distance. Raw DNA methylation data were processed with default pre-processing steps in UniD (15) as implemented in R. Samples with more than 10% of values missing and probes with more than 3 missing values were excluded. The isocitrate dehydrogenase (*IDH*) mutation status for each sample was predicted using UniD predictive models (15). The methylation level was represented as a β value [methylated signal divided by the sum of methylated and unmethylated signal (16)]. We first applied unsupervised clustering and t-distributed stochastic neighbor embedding (t-SNE) (17) analysis with the top 200 and 500 probes with the highest median absolute deviation (MAD) values across all samples. Then, using only the most variant probes, we removed probes that are likely uninformative and only reduce statistical power (18). In order to calculate the methylation distance between biopsies pairs from the same patients, we used the top 500 probes with highest variance within each patient to calculate the L_1_ distance:

$$\begin{array}{l} d_{L1}=\underset{i}{\sum }\left|P_{i}-Q_{i}\right| \end{array}$$

Where *P*_*i*_ is the *i*th beta value of the first sample in the pair and *Q*_*i*_ is the beta value of the second sample in the pair (12). The L_1_ distance metric measures the total variation in methylation values and identical profiles have zero distance. Since the beta values are already normalized as the sum of methylated and unmethylated signal, we used the standard L_1_ distance metric rather than the Canberra distance used for CNV genetic distance. The correlation between the methylation distance and Euclidean distance was measured with the Pearson correlation coefficient.

## Results

**Figure 1:**
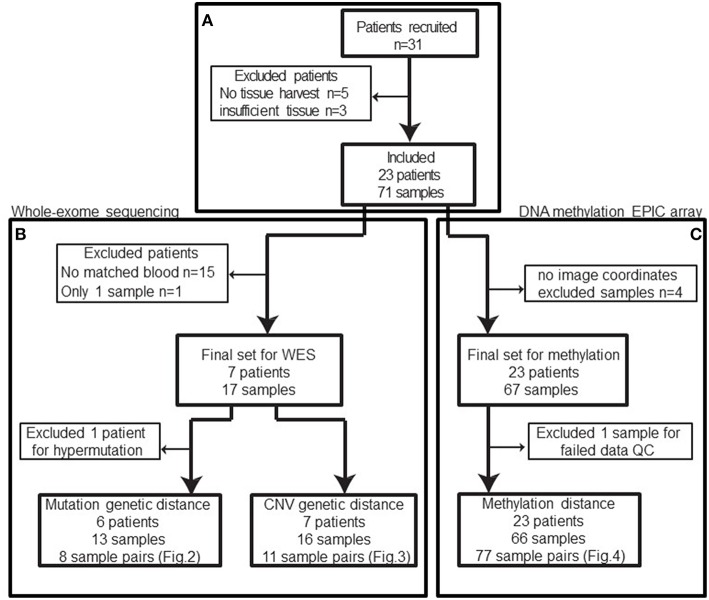
Patient and samples cohort processing flowchart. **(A)** 31 patients were recruited between 2013 and 2016. Five patients had no tissue harvest due to surgical complexity (*n* = 3), cardiac issues, or technical difficulties and three patients had no sufficient tissue. In total, eight patients were excluded from the cohort. **(B)** Among the remaining patients, only eight patients had normal blood samples available for whole-exome sequencing and one of those patients only had a single sample sequenced. Based on the mutation calls, one patient was excluded due to abnormally high mutation burden (TMB). The remaining 13 biopsies from 6 patients constitute 8 biopsy pairs for mutation genetic distance. **(C)** After excluding samples without image coordinates, we have 67 samples from 23 patients for methylation profiling. One sample was excluded from methylation analysis due to poor data quality: 12.85% of available probes returned missing values. In summary, 66 samples from 42 unique image guided biopsy sites in 23 patients were available for methylation data analysis, comprising 77 unique biopsy pairs. The specific patients included in each analysis is available in Table 1.

In total, 31 patients were recruited between 2013 and 2016. Patients with no tissue harvest due to surgical complexity, cardiac issues, or technical difficulties or patients with insufficient tissue for downstream molecular analysis were excluded from our study cohort (*n* = 8) (Figure 1A) leaving 23 patients with 71 biopsies.

After exclusions (4 samples) for ambiguous imaging coordinates, 67 samples from 23 patients were subjected to global methylation array-based profiling (Figure 1C). Seventeen of these samples from 8 patients were processed for WES (Figure 1B). These patients were selected because patient-matched blood samples were retrospectively available for somatic DNA assessment thanks to an institutional tumor banking initiative. A summary of the patient demographic information is given in Table 1. Of the 67 total samples in the final analysis, 46 samples were shallow/deep pairs from needle biopsy, 4 were single samples from needle biopsy, 15 were single samples from forceps biopsy, and 2 were a shallow/deep pair collected from the same spatial location using forceps.

**Table 1:** Patient demographic information.

**Patient information**				**Clinical information**						**Test applied**	
**Pt #**	**# biopsy samples**	**# blood samples**	**# sample pairs**	**Age**	**Sex**	**Primary diagnosis**	**WHO**	**1p/19q status**	**IDH1**	**EPIC**	**WES**
1	3	1	3	36	F	OA	II	Codel	WT	Yes	Yes
2	2	0	1	25	F	Anaplastic Diffuse Glioma	III	Codel	Mut	Yes	No
3	2	0	1	21	F	Anaplastic Diffuse Mixed OA	III	Codel	Mut	Yes	No
4	6	0	10	26	F	GB	IV	Neg	Mut	Yes	No
5	4	0	6	75	F	Diffuse Astrocytoma	II	Neg	WT	Yes	No
6	2	0	1	56	F	Diffuse Glioma	II	Neg	Mut	Yes	No
7	4	0	6	54	F	GB	IV	Neg	WT	Yes	No
8	4	0	6	45	M	Anaplastic Astrocytoma	III	Neg	WT	Yes	No
9	4	0	6	28	M	OD	II	Codel	Mut	Yes	No
10	3	0	3	30	F	Anaplastic Astrocytoma	III	Neg	Mut	Yes	No
11	2	0	1	62	M	GB	IV	Neg	WT	Yes	No
12	3	1	3	80	M	GB	IV	Neg	WT	Yes	Yes
13	2	0	1	44	M	Anaplastic Astrocytoma	III	Neg	Mut	Yes	No
14	6	0	15	55	F	OD	II	Codel	Mut	Yes	No
15	2	1	1*	67	M	GB	IV	Neg	WT	Yes	Yes^*^
16	2	1	1	32	M	OD	III	Codel	Mut	Yes	Yes
17	2	1	1	66	M	Diffuse Astrocytoma, GB	IV	Neg	Mut	Yes	Yes
18	2	0	1	41	F	Anaplastic OD	III	Codel	Mut	Yes	No
19	2	0	1	58	F	Diffuse Astrocytoma, GB	IV	Neg	WT	Yes	No
20	2	1	1	35	F	OD	II	Codel	Mut	Yes	Yes
21	2	1	1	49	F	GB	IV	Neg	WT	Yes	Yes
22	2	1	1	32	M	Anaplastic Astrocytoma	III	Neg	Mut	Yes	Yes
23	4	0	6	39	F	Diffuse Astrocytoma	II	Neg	Mut	Yes	No

List of each patient included in the final analysis. EPIC indicates DNA methylation EPIC array was performed on that patient's samples (for methylation genetic distance) and WES indicated whole-exome sequencing (for mutation and copy number variation genetic distance). Sample pairs refers to the number of biopsy sample pairs that were available to calculate spatial and genetic distance. Patient age, sex, primary diagnosis, WHO grade, 1p/19q, and IDH mutation status are listed for reference. GB, Glioblastoma; OD, oligodendroglioma; OA, oligoastrocytoma. *WES only applied to one of two samples due to insufficient tumor content in one sample. EPIC methylation assay was performed on both samples.

Genomic and physical distances were only calculated on an intra-tumor basis, meaning samples were not compared between patients, but only to other samples in the same patient/tumor.

### Mutation Count Genetic Distance

WES was performed to identify gene mutations in the biopsy tissue samples. The mean coverage was 117 and 103 for tumor tissues and WBCs, respectively. We used the MuTect2 (10) and ANNOVAR (11) for somatic mutation calling and annotation. After filtering mutations, we identified a total of 257 single nucleotide variants (SNVs) and 19 insertions/deletions (indels) in our final 14 tissue samples analyzed.

In further examining the profiles of three biopsies (P12S1, P12S2, and P12S3) from one patient (patient 12), we found significantly higher mutation calls than in the other patients. Even after applying mutation filters, we found patient 12 had on average 2438 mutations per biopsy while all other patients/samples averaged 22 mutations per biopsy. In published literature (19), the median mutation rate per million base (Mb) is <1 for lower grade gliomas. So, the median mutation number for the whole exome (about 30 Mb) is <30. Therefore, we believe these three biopsies show hypermutation. We eliminated the possibility of a mismatched blood sample by comparing the non-conserved long insertion sequence between the blood and tumor samples, which were found to be consistent. Further review of this case revealed a prominent history of cancer in the patient's family, suggesting a fundamentally distinct mechanism of tumor evolution from those utilized in the remaining cohort. For these reasons, we excluded these samples from mutation count genetic distance analysis (Figure 1B), leaving 14 biopsies in 7 patients for analysis.

**Figure 2:**
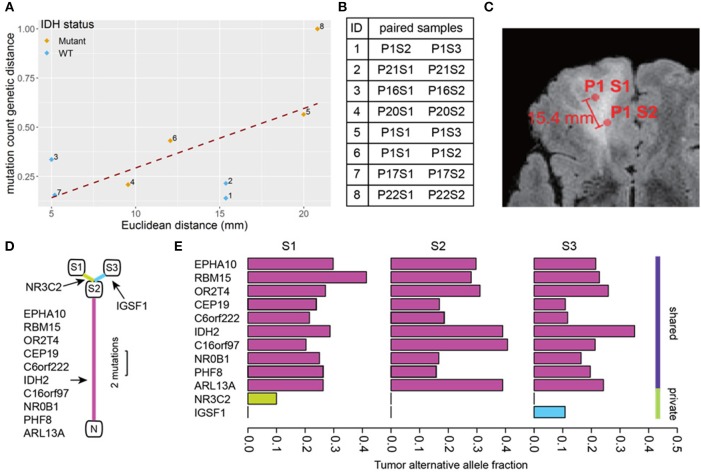
Mutation count genetic distance. **(A)** Scatter plot shows a high correlation between Euclidean distance and the Jaccard distance between biopsy pairs (Pearson *r* = 0.63). **(B)** The pairs of biopsy points whose distances are graphed in **(A)**. The samples are listed by patient number (P) and sample number (S) so for example pair 1 consists of sample 2 from patient 1 (P1S2) and sample 3 from patient 1 (P1S3). **(C)** Physical distance illustration. On this magnetic resonance image, a biopsy pair in patient 1 (samples P1S1, P1S2) are indicated by circles. The Euclidean distance between the sample sites is shown. **(D)** The phylogenetic tree of three biopsies from the same patient (P1). N represents normal brain (no mutations), and S1-3 are the three biopsy sites sampled. The detected mutations events are shown in the annotations, the segment length is proportional to the number of mutations. **(E)** The tumor alternative allele fraction for all mutation events were compared between biopsies shown in **(D)**. The shared mutations between all samples generally show a higher alternative allele frequency.

A total of 74 somatic mutations identified in our initial WES were then validated by focused Ion Proton sequencing, yielding a concordance rate of 100% (74/74 mutations, Supplementary Table S1 with primer sequences in Supplementary Table S2). Confident in the quality of our sequencing data, we proceeded to determine the genetic distance as measured by mutation count between patient-matched samples for our remaining pairs as a function of number of distinct mutations. Similar approaches have been applied in recent work (20). We then correlated mutation count genetic distance to Euclidean distance and found a strong correlation (Pearson correlation coefficient = 0.63, *p* = 0.091) (Figure 2), supporting the notion that as the physical distance between biopsy samples increases, so too does the number of mutated genes. Indeed, some of the most closely clustered samples (Patient 1) by Euclidean distance (5 mm) exhibited only one distinct mutation whereas two samples biopsied 21 mm apart (Patient 22) had 36 distinct mutations between them. On average a one unit increase in mutation count genetic distance unit was equivalent to an increased Euclidean distance of 0.6 mm, and 10 mm of additional Euclidean distance was equivalent to 17 additional mutation counts. The equation of the best fit regression line was: Genetic count distance = −11.6 + 1.7· Euclidean distance in mm.

Using the three samples from patient 1 as unique samples for further exploration, the hierarchical structure between biopsies was investigated (Figure 2). By comparing mutation calls among biopsies, we found that all three samples shared 10 common mutations, while P1S1 or P1S3 each had one additional distinct mutation (Figure 2). Finally, the allele frequency of mutation calls (Figure 2) were generally higher for the shared mutations between the three samples than for the private mutations, suggestive of sub-clonality within independently evolving tumor clones.

### Copy Number Variation Genetic Distance

**Figure 3:**
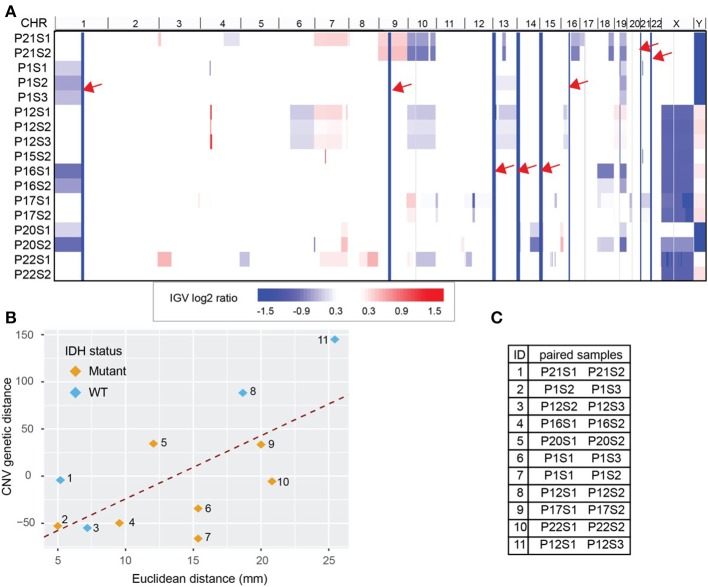
Whole Exome Sequencing (WES) derived copy number variations (CNV) distance. **(A)** CNV shown in Integrative Genomics Viewer (IGV). Chromosomes are labeled at the top of the panel and sorted in order from chromosome 1 to chromosome Y. Each row represents one sample identified by patient number (P) and sample number (S). The color blocks show the CNV log2 ratio value: blue indicated loss of copies while red indicated amplification. For regions with the same CNV across samples (solid column of blue marked with red arrows) there is no information across all samples. **(B)** CNV distance showed high correlation with the Euclidean distance between biopsy pairs from the same patient (Pearson *r* = 0.65). Pairs were drawn with color indicating IDH mutation status. **(C)** The paired sample details based on the label in **(B)**. Each sample is labeled by patient number (P) and sample number (S).

Copy number variation (CNV) for each biopsy was derived from WES data using CNVkit and visualized with Integrative Genomics Viewer (IGV, version 2.4.8) (21, 22) (Figure 3A). We obtained 255 CNV events after combining all break points available. WES data also estimated cellularity to be >50% for a majority of samples used in CNV analysis (Supplementary Table S3). Reassuringly, we found that our data recapitulated well-known glioma-associated patterns such as 1p/19q co-deletion and co-incident 7-gain/10-loss, characteristic of IDH-mutant oligodendroglioma and IDH-wild type glioblastoma, respectively (Table 1).

Using the log2 ratio value as input, CNV distance was calculated between each biopsy pair. Since the algorithm inferring CNV using WES data relies on the read counts instead of mutation calls, we included the hypermutated case of patient 12 in our CNV analysis (Figure 3C). We compared CNV distance with Euclidean distance for each paired set of biopsy specimens and once again obtained a strong correlation (Pearson correlation coefficient = 0.65, *p* = 0.04, Figure 3B). Moreover, linear regression between CNV distance and Euclidean distance (slope constant was approximately 6.8 log2 CNV per mm) showed the same trend as was seen between mutation count genetic distance and Euclidean distance. IDH mutant and wild-type samples both demonstrated the same general relationship between CNV distance and Euclidean distance. On average 10 mm additional distance increased the CNV distance by 68.4 units. Each unit of CNV distance corresponded to about 0.15 mm Euclidean distance. The equation of the best fit regression line is: CNV distance = − 93.8 + 6.9· Euclidean distance in mm.

### Methylation Genetic Distance

**Figure 4:**
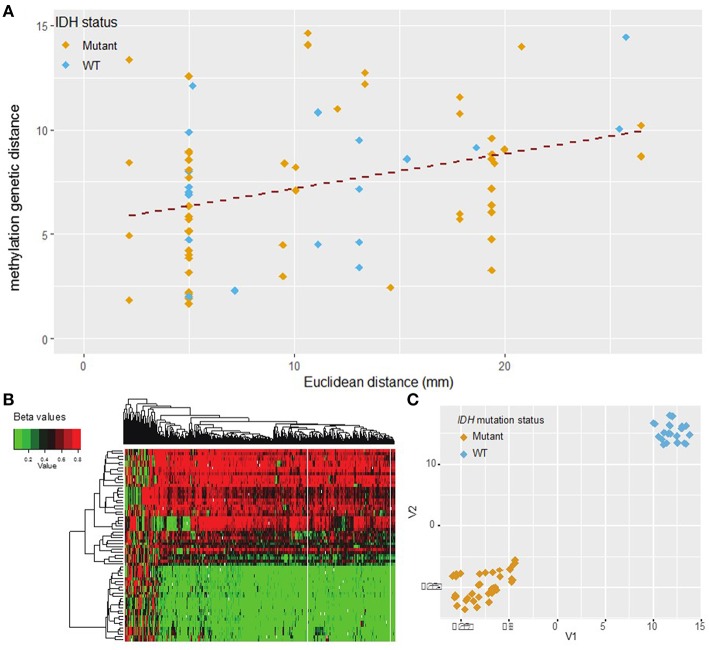
DNA methylation L_1_ distance vs. Euclidean distance. **(A)** The L_1_ distance measures total variability in the methylation profile. This measure shows moderate correlation with Euclidean distance (Pearson *r* = 0.35, *p* = 0.002). Shallow/deep pairs from the same biopsy sample are assumed to be 5 mm distant. **(B)** Heatmap of hierarchical clustering with the top 500 probes with highest median absolute deviation (MAD) values. Each row represents one sample and each column one probe. **(C)** t-SNE plot of the top 500 probes with highest MAD values (probes with missing values were removed). The marker color indicates IDH mutation status. A natural clustering into IDH mutated and wild type tumors is evident in both the heatmap and t-SNE plot.

After data pre-processing with the UniD algorithm (see materials and methods), one sample was excluded due to high probe fail percentage (>10%) (Figure 1). Unsupervised hierarchical clustering of the remaining 500 probes and 66 samples delineated two subgroups within the cohort as evidenced by a heatmap (Figure 4, Supplementary Figure S1). The strong separation of the two clusters was further illustrated by t-SNE analysis and visualization (Figure 4). The composition of the two clusters showed a well-established concordance to *IDH* mutational status (23) (Supplementary Table S4).

Methylation distance was then independently calculated between all possible biopsy pairs from each patient using the L_1_ distance between the values of the top 500 most variant methylation probes. Comparing these findings with Euclidean distance once again revealed a significant correlation (Pearson correlation coefficient 0.35, *p* = 0.002) (Figure 4). The abundance of sample pairs at a Euclidean distance of 5 mm is due to the shallow and deep portions of the same biopsy specimen, separated from each other by 5 mm, being analyzed separately (Supplementary Figure S2). The methylation distance between these shallow/deep pairs spanned the entire dynamic range, a finding not seen for other measures of molecular distance (see above). This discrepancy may be due to greater fluctuation in the DNA methylation profile between samples compared to mutational or copy number variation or may be due to the increased number of samples available for methylation analysis. Regardless, there is a significant correlation (correlation coefficient = 0.35, *p* = 0.002) between methylation distance and Euclidean distance and the minimum methylation distance between samples increased substantially with a Euclidean distance above about 2 cm. Based on the best-fit regression line we estimate an increase in the methylation distance of about 1.8 per 10 mm Euclidean distance, with each unit of methylation genetic distance corresponding to about 5.6 mm of Euclidean distance. The best-fit regression line equation was: Methylation genetic distance = 5.27 + 0.18·Euclidean distance in mm. The relation between methylation genetic distance and Euclidean distance is fairly consistent between samples from IDH wild-type and IDH mutant tumors as seen visually in Figure 4. The correlation remains statistically significant even when only samples with similar IDH mutation or 1p/19q co-deletion status are considered. See the Supplementary Figures S3, S4 for details.

## Discussion

Many recent studies have documented the heterogeneity characterizing malignant glioma (24–26). Delineating the molecular mechanisms driving this heterogeneity remains an active area of investigation, as does the optimization of techniques for its non-invasive assessment. In this study, we aimed to establish informative and quantitative links between heterogeneity and spatial distance in a small glioma patient cohort. Among the most basic measures of spatial variability is simple Euclidean distance, and we found strong correlations between this metric and multiple assessments of molecular distance for distinct genomic/epigenomic variables. Two of these “molecular distances” were based on some form of total variation, or L_1_ distance, an additional similarity, and the third (mutation count genetic distance) used a sum-of-squared distances. Future work may incorporate image data to develop a more complex measure of “radiographic distance” to complement physical distance. Our findings confirm prior work showing that gliomas exhibit spatial variability in their genomic signatures dependent on precise biopsy site location (5, 6, 24, 27). Moreover, they establish, for our limited patient population, a set of correlation constants for the various measures of molecular distance and position in Euclidean space. The fact that three distinct molecular features, (1) somatic mutations, (2) CNVs, and (3) global methylation profiles, tracked similarly with Euclidean distance is notable.

Similar work includes a study conducted recently by Lee et al. (5), where the authors calculated Nei's genetic distance in multisector samples of glioblastomas and found that this metric was greater for samples that were farther apart in space (i.e., distant vs. local recurrence). Note: the Nei's distance analyzes genetic variability within populations which is not applicable to our sample size, hence why we used other distance measures in our analysis. Additional work by Sottoriva et al. analyzed copy number and gene expression data from multiple samplings of glioblastomas to illustrate how tumor phylogeny can be related to the approximate spatial position (24). Our study is consistent with these earlier reports. In addition, we measure the actual physical distance between samples and demonstrate a significant linear relationship between spatial and molecular distance in glioma. This correlation suggests that the processes of molecular and spatial evolution in tumor cells may be fundamentally linked. A proposed mechanism for tumor heterogeneity is that distinct molecular characteristics become apparent in cancer cell clones as they distribute themselves across a given tumor mass over time (28, 29). While our present study does not investigate this mechanism directly, it is one potential explanation for the correlation between spatial and genetic distance. Whether acquired molecular alterations actively drive cellular motility as a rule, however, remains less certain. Recent literature suggests that branching mutational profiles of multiple tumor samples are due in part to differences in selective pressures (6, 7, 29), from environmental factors such as hypoxia (30). Such constraints could fundamentally drive molecular evolution as a means to escape suboptimal microenvironments. However, simple expansion of a tumor mass would also be expected to passively drive clones apart that, over time, would acquire increasing molecular distinctiveness.

This proposed mechanism does not account for hypermutated cases such as the patient we discussed previously. Given that the patients in our study were previously untreated, we can exclude the possibility of these mutations being caused by alkylating chemotherapeutic agents. In the absence of prior treatment the hypermutation status suggests an underlying germ line mutation, although our analysis precludes certainty. This is further supported by the patient's strong family history of cancer (31, 32).

Although our results show substantial differences in the number of mutations between samples from the same patient (Supplementary Table S5), we found that some root-level carcinogenic mutations like IDH1 were consistently present or absent in all samples from a given patient (Supplementary Table S4). Genomic findings thus support a branched evolution pattern, where some genetic events, in particular IDH1 mutations, are fundamental, required for tumorigenesis and are thus present in all samples. Accordingly, these alterations are truncal, with more unusual mutations relegated to sub-clonal events in selected populations (5, 6). These early, required mutations, tend to be diagnostically important, as reflected by the inclusion of IDH1 in the WHO grading criteria (1). These findings also reflect multiple published reports on clonal evolution within malignant glioma (5, 27).

As the classification and prognosis of gliomas is substantially influenced by genomic features, we suspect that the specific relationship between spatial and molecular distance might depend on the grade and type of the glioma. Within our sample set, additional subgroupings could be made based on established and prognostically relevant molecular stratifiers such as IDH mutation status and MGMT promotor methylation. However, our patient population is not large enough to examine distinctions within these smaller subgroups with sufficient statistical power. Nevertheless, our results using a combined glioma population across grades and subtypes suggests the positive and linear relationship between spatial and genetic distance is a characteristic of gliomas in general.

The concept of genetic heterogeneity is not novel, but our work is the first attempt (to our knowledge) to formally quantitate the relationship between spatial and genetic distances. We chose to use the simplest measure of correlation between spatial and genetic distance (i.e., linear) as the initial avenue of investigation. More complex methods of quantitating this relationship in the future may provide better correlation or interpretability. We also look to future investigations to elucidate the undoubtedly complex relationships between glioma subtypes, grades, and diverse genomic selectors, and the spatial distribution of genomic heterogeneity.

We propose that the further exploration of such genomic-spatial relationships in clinical trials similar to the current study, is justified. Establishing first the fundamental, and later on, more sophisticated imaging-genomic correlates, will put the field of imaging genomics on a firm scientific footing, and develop it into something that could be made useful for patient care.

## Conclusion

The genetic heterogeneity of gliomas is correlated to physical distance within individual tumors, as confirmed by quantitative relationships using multiple independent methods. These findings likely support a diverging clonal evolutionary model of glioma expansion.

## Data Availability

The datasets for this manuscript are not publicly available because analysis as part of a larger study is still ongoing. Requests to access the datasets should be directed to DS, dawid.schellingerhout@mdanderson.org.

## Ethics Statement

This study was carried out in accordance with the recommendations of The University of Texas MD Anderson Cancer Center IRB with written informed consent from all subjects in accordance with the Declaration of Helsinki. The protocol was approved by The University of Texas MD Anderson Cancer Center IRB.

## Author Contributions

EG, JY, KF, JL, JW, SP, DF, ES, JH, and DS: study design. EG, JY, KF, JW, SP, LL, DF, JH, and DS: data collection, analysis, and interpretation. EG, JY, KF, JL, DF, ES, JH, and DS: manuscript writing. EG, JY, JH, and DS: figures.

### Conflict of Interest Statement

The authors declare that the research was conducted in the absence of any commercial or financial relationships that could be construed as a potential conflict of interest.

## Footnotes

^1^https://github.com/JieYang031/WGS-analysis
