# Supplementary material for: Spatial Distance Correlates With Genetic Distance in Diffuse Glioma
Source: Front Oncol. 2019 Jul 30;9:676. doi: 10.3389/fonc.2019.00676 (PMC6682615; doi:10.3389/fonc.2019.00676)
Supplement: Supplementary file 1 [file Data_Sheet_1.docx]

**Spatial Distance Correlates with Genetic Distance in Diffuse Glioma**

Supplementary Material

# Supplementary Tables

See separate data file for supplementary tables S1 – S5

# Supplementary Methods

## DNA methylation clustering analysis

With the same DNA methylation data set used in the methylation distance calculation, the MAD value was calculated across all samples for each probe. The top 200 probes with highest MAD value were selected and subjected to hierarchical clustering with default settings. 172 probes after probes with missing values were removed. We then applied T-distributed stochastic neighbor embedding (TSNE) and compared the subgroups memberships between hierarchical clustering and TSNE.

## WES processing

For all WES fastq files, fastqc was applied for quality control purpose before alignment. BWA MEM was used for alignment, followed with mark duplicate reads, identify realign targets, realign insertion and deletion, and recalibrate values. GATK was used for coverage checking. After mapping, Mutect2 was applied to call variants using default setting. ANNOVAR was used to annotate mutation calls.

# Supplementary Results

## DNA methylation clustering analysis

With the top 200 probes, hierarchical clustering analysis showed that samples also separated into two different subgroups, named as cluster 1 and cluster 2 (**Figure S1B-C**). These clusters are very similar to the clusters seen in Figure 4 using the top 500 probes, and the memberships show high concordance.

# Supplementary Figures


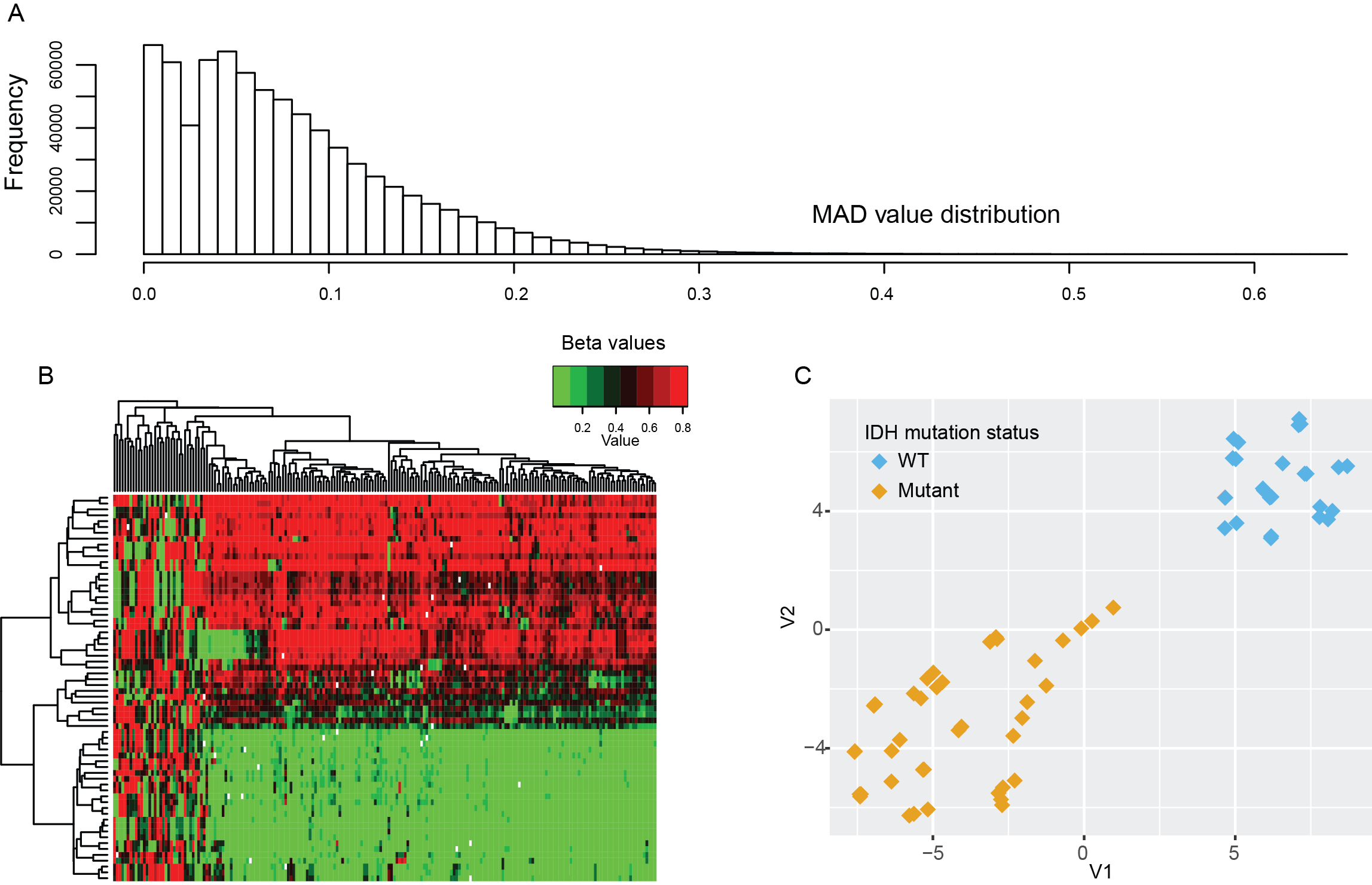


**Supplementary Figure S1.** **DNA methylation clustering analysis.** A: Median absolute deviation (MAD) value distribution for all probes. The number of probes decreases roughly exponentially with MAD above about 0.05. B: Heatmap of hierarchical clustering with the top 200 probes with highest MAD values. Each row represents one sample and each column one probe. The order of the samples are the same as in supplementary. C: TSNE plot of the top 200 probes with highest MAD values (probes with missing values were removed). The marker color indicates IDH mutation status. Note the slightly weaker clustering by IDH status than what is seen in Figure 4C using 500 probes.

**
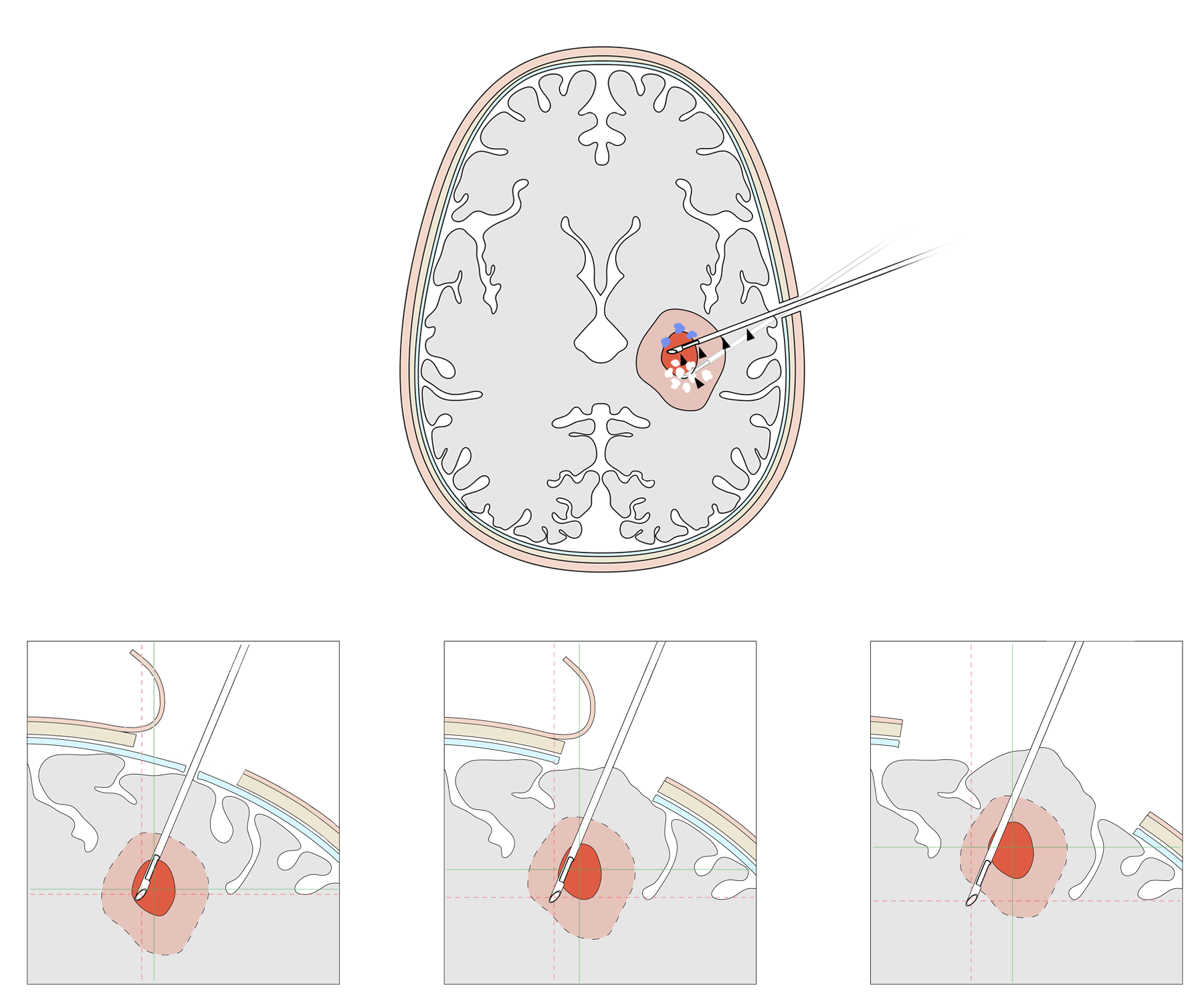
**

**Supplementary Figure S2: Illustration of the Nashold biopsy needle used to collect biopsy specimens.** The surgical navigation software is correlated with the tip of the instrument (red dashed lines). The distance from the tip to the center of the side window is 7 mm and this distance is used to obtain the sample coordinates at the time of collection. For several specimens, the cylindrical core samples was cut in half for separate analysis of the “shallow” and “deep” ends. The ends were assumed to be 5mm distant. Note the samples are collected prior to the opening of the dura (blue) to avoid brain shift.


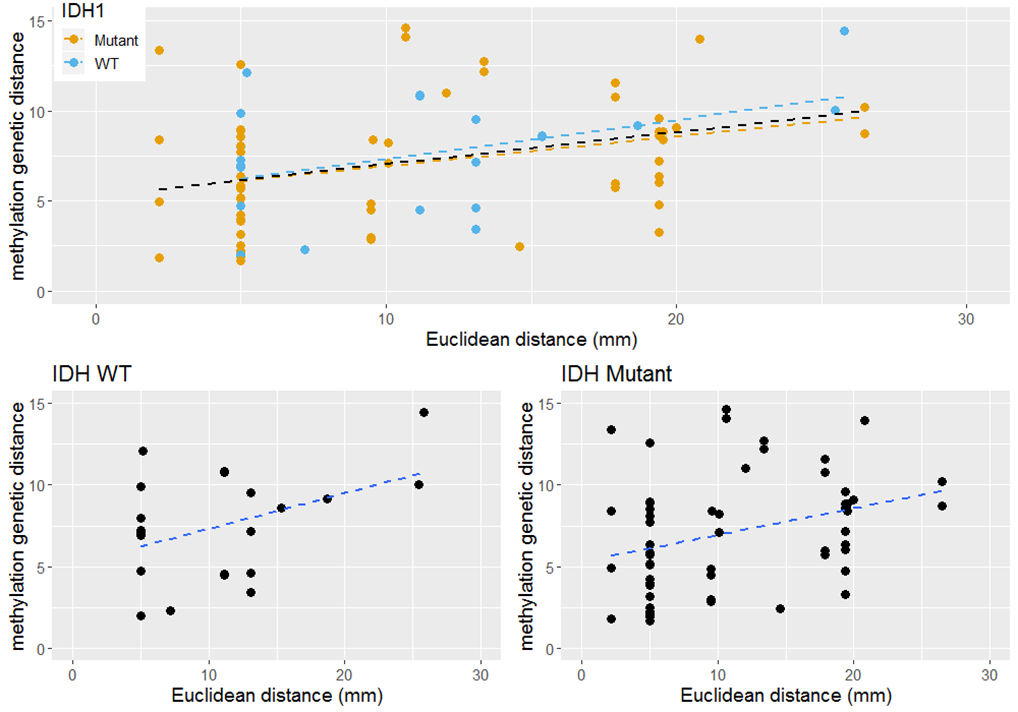


**Supplementary Figure S3: Methylation genetic distance by IDH status.** Top: recreation of Figure 2 with best-fit lines plotted for the combined data (black), only IDH1 mutant sample pairs (orange), and only IDH WT pairs (blue). The data are plotted separately in the bottom panels. The correlations for the subset data are very similar (r = 0.43 for IDH WT, 0.32 for IDH Mutant, 0.35 combined) and the trends are significant (p < 0.05) in all cases.


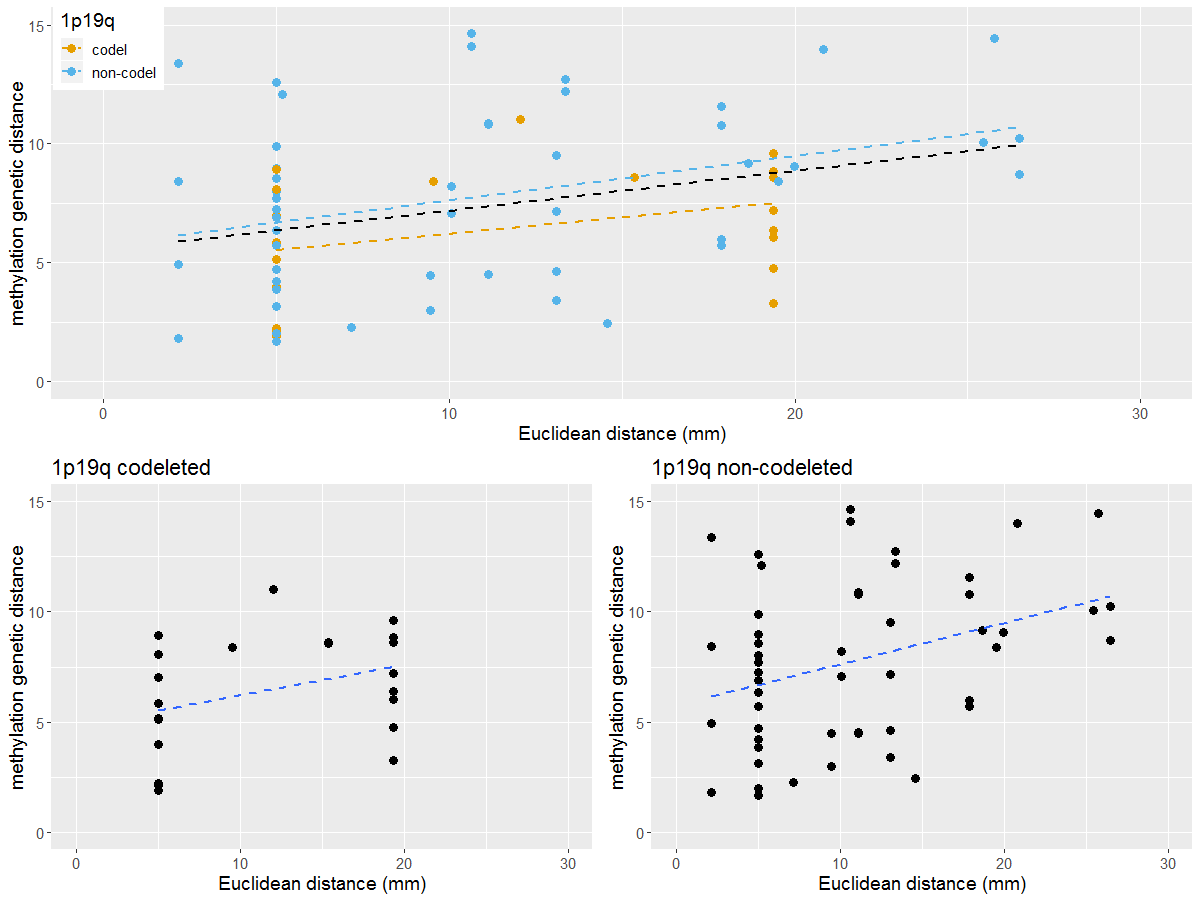


**Supplementary Figure S4: Methylation genetic distance by 1p/19q codeletion status.** Top: recreation of Figure 2 with best-fit lines plotted for the combined data (black), only 1p/19q codeleted samples (orange), and only non-codeleted samples (blue). The data are plotted separately in the bottom panels. The correlations for the subset data are very similar (r = 0.36 for 1p/19q codeleted, 0.35 for non-codeleted, 0.35 combined). The trend remains significant with p < 0.05 for the non-codeleted samples as well.

# Supplementary Table Captions

**Table S1: Validation results for WES by focused Ion Proton sequencing.** The concordance rate for the validated samples (P17S1, P21S2, and P22S2) is 100%.

**Table S2: Primer sequences for WES**. Sequences are listed for the three samples (P17S1, P21S2, and P22S2) used for validation of WES.

**Table S3: Estimated tumor content.** Using CNV data we estimated the cellularity and ploidy of each sample. Cellularity was >50% indicating substantial tumor content.

**Table S4: Methylation profile cluster data.** Samples were clustered using t-SNE analysis and the clusters correspond strongly to IDH mutation status. For each sample, the IDH mutation status is listed in three ways: as given in the patient’s clinical record, as predicted by methylation analysis, and as found by WES (if available). The only discordance is in the case of IDH2 mutation which is not tested for clinically but will be detected by methylation profiling or WES.

**Table S5: Euclidean and genetic distance data.** For each pair of samples the physical (Euclidean) distance between samples is listed along with the three measures of genetic distance. Jaccard genetic distance (mutation genetic distance), CNV genetic distance, and methylation genetic distance are plotted in Figures 2-4. For reference the clinical IDH1 mutation status, 1p/19q co-deletion status, and MGMT promotor methylation are also given for each sample pair.
